# Preliminary findings on left atrial appendage occlusion simulations applying different endocardial devices

**DOI:** 10.3389/fcvm.2023.1067964

**Published:** 2023-02-20

**Authors:** Nadia D’Alessandro, Matteo Falanga, Alessandro Masci, Stefano Severi, Cristiana Corsi

**Affiliations:** Department of Electrical, Electronic and Information Engineering, University of Bologna, Bologna, Italy

**Keywords:** atrial fibrillation, computational fluid dynamics, left atrium, left atrial appendage occlusion, left atrial appendage

## Abstract

Atrial fibrillation (AF) is one of the most investigated arrhythmias since it is associated with a five-fold increase in the risk of strokes. Left atrium dilation and unbalanced and irregular contraction caused by AF favour blood stasis and, consequently, stroke risk. The left atrial appendage (LAA) is the site of the highest clots formation, increasing the incidence of stroke in AF population. For many years oral anticoagulation therapy has been the most used AF treatment option available to decrease stroke risk. Unfortunately, several contraindications including bleeding risk increase, interference with other drugs and with multiorgan functioning, might outweigh its remarkable benefits on thromboembolic events. For these reasons, in recent years, other approaches have been designed, including LAA percutaneous closure. Unfortunately, nowadays, LAA occlusion (LAAO) is restricted to small subgroups of patients and require a certain level of expertise and training to successfully complete the procedure without complications. The most critical clinical problems associated with LAAO are represented by peri-device leaks and device related thrombus (DRT). The anatomical variability of the LAA plays a key role in the choice of the correct LAA occlusion device and in its correct positioning with respect to the LAA ostium during the implant. In this scenario, computational fluid dynamics (CFD) simulations could have a crucial role in improving LAAO intervention. The aim of this study was to simulate the fluid dynamics effects of LAAO in AF patients to predict hemodynamic changes due to the occlusion. LAAO was simulated by applying two different types of closure devices based on the plug and the pacifier principles on 3D LA anatomical models derived from real clinical data in five AF patients. CFD simulations were performed on the left atrium model before and after the LAAO intervention with each device. Blood velocity, particle washout and endothelial damage were computed to quantify flow pattern changes after the occlusion in relation to the thrombogenic risk. Our preliminary results confirmed an improved blood washout after the simulated implants and the capability of foreseeing thrombogenic risk based on endothelial damage and maximum blood velocities in different scenarios. This tool may help to identify effective device configurations in limiting stroke risk for patient-specific LA morphologies.

## 1. Introduction

Atrial Fibrillation (AF) is the most common form of arrhythmia worldwide with a prevalence of almost 20% in patients aged 80 years or older and lower prevalence but still non-negligible (about 5%) in younger population. It is considered the “new cardiovascular disease epidemic of our century” and estimates predict that 12 million people in the United States will have AF by 2030 (1). It is characterized by an irregular, disorganized and very rapid heart rhythm inducing, in long term, structural and functional changes (2). Based on these epidemiological data (1, 3), this arrhythmia remains one of the major causes of stroke, heart failure, sudden death, and cardiovascular morbidity in the world and represents a major clinical, social and economic burden (4, 5).

Scientific evidence shows AF is an independent risk factor for stroke: AF patients suffer from a five-fold increased risk of cerebrovascular events (6, 7), being responsible of 15–18% of all strokes. Structural remodeling of the LA includes a progressive main chamber enlargement (8) and left atrium appendage (LAA) elongation (9). Such changes lead to a mechanical function adaptation causing a chaotic and strongly reduced contractile activity. These modifications also affect the physiological hemodynamics within the LA, contributing to blood stasis, clot formation and embolism. In addition, because of its finger-like morphology, the LAA is the left atrial site of the highest blood stasis risk, increasing the incidence of thrombus formation and stroke: it was reported 90% of intracardiac thrombi in patients with cardioembolic stroke/transient ischemic attack (TIA) are originating in the LAA (10).

Treatments to limit stroke risk include oral anticoagulation (OAC) therapy which was the only option available until recently. Unfortunately, several contraindications including bleeding risk increase, interference with other drugs and multiorgan functioning, might outweigh its remarkable benefits on thromboembolic events (11). For these reasons, in recent years, other approaches have been designed, including LAA percutaneous closure, which seems to better reduce the risk of thromboembolism compared to warfarin anticoagulation therapy (12). Unfortunately, nowadays, LAA occlusion (LAAO) is restricted to small subgroups of patients being associated with procedural risks and costs which may overcome the preventive antiembolic efficacy. Several trials have shown that left atrial appendage occlusion (LAAO) is effective and not inferior to oral therapy in stroke prevention (13–15). The multicenter EWOLUTION European registry reported a high rate of implantation success (98%), with an acceptable procedure-related complication rate of 4% at 30 days follow-up (16). However, additional controlled trials are urgently needed to define the best use of the occlusion devices in patients unsuitable for oral anticoagulants (OAC) or suffering a stroke on OAC; randomized comparisons of LAAO with new OACs (NOACs) are also missing, as well as an objective assessment of the minimal antiplatelet therapy acceptable after LAAO.

Since the LAAO device implantation can cause serious complications and major adverse events, the utmost attention must be paid when clinical treatment decision-making is performed balancing benefits and risks linked to this procedure (17–19) Although mortality rate is around 3% considering a mean of all the studies presented in literature on this topic, other adverse events can occur. Indeed, the two most important complications that occur with LAAO are peri-device leaks and device-related thrombus. Such complications have become an important concern because of their incidence and the increased rate of associated stroke (20). In addition, pericardial effusion, stroke or TIA after the implantation procedure were also observed. Bleeding around the device, device embolism, device motion and dislocation have been detected. Based on these considerations, the LAA percutaneous closure is a not trivial procedure that requires a certain level of expertise and training to successfully complete the procedure without complications.

Moreover, the anatomical variability of the LAA plays a key role in the choice of the correct LAA occlusion device and in its correct positioning with respect to LAA ostium during the implant. To take into account the anatomical variability, LA cavity is modeled using biomedical images, before and during the intervention. The most used acquisition modalities in clinical centers are X-ray and transesophageal echocardiography (TEE), being able to characterize the LAA morphology during the intervention to support decisions on device implantation. The LAA ostium dimensions and height/depth of the LAA cavity are critical LAA shape parameters to define the optimal size of the device to be implanted and of the landing zone. Until recently, such parameters were estimated from medical images applying manual tools based on the operator expertise, not being available standardized criteria to objectively define them. In addition, values of these parameters estimated from different imaging modalities differ substantially due to their respective spatial resolution and limitations. In recent years, studies have been published on computational models for virtual implantation of left atrial appendage devices, intended as a useful tool for clinicians to optimize LAAO preprocedural planning for patient-specific anatomies ((21, 22)).

For hemodynamics assessment, in clinical practice, the analysis is commonly based on Doppler echography; however, Doppler imaging only reports a 2D blood flow velocity profile (e.g., LAA ostium) in time, which constitutes an over-simplification of the complex 3D hemodynamics in the LA and LAA.

In this scenario, hemodynamics plays a very important role and computational fluid dynamics (CFD) could provide a key contribution for *in silico* simulations of the blood flow patterns after the occlusion on a patient-specific basis to assess flow stagnation.

Only few studies focused on the hemodynamic changes in LA pre- and post-LAAO (21, 23); in most studies (24–27) the hemodynamic effects of the closure were assessed in terms of blood flow, endothelial cell activation potential (ECAP) and device-related-thrombosis (DRT). Regarding left atrial fluid simulations, a nice review of boundary conditions and different modeling choices is reported in (26). Only few studies took into account contraction in AF condition: rigid walls, contraction derived by dynamic CT or MRI in sinus rhythm and diffusion-based dynamic mesh considering only passive movement of the LA produced by the contraction of the left ventricle are the most spread choices (26).

The aim of this study was to simulate the fluid dynamics effects of the LAA occlusion in AF patients to predict hemodynamic changes caused by the LAAO. LAAO was simulated by applying the two different types of occluders available on the market based on the plug and on the pacifier principles (28), on 3D LA anatomical models derived from real clinical data in five AF patients. CFD simulations were performed on the left atrium model before and after the LAAO intervention and in AF condition; fluid dynamics indices including blood velocity, particle washout, endothelial damage and device-related-thrombosis were computed to quantify flow pattern changes after the occlusion in relation to the thrombogenic risk.

## 2. Methods

### 2.1. Patients data and LA models creation

The computational domain of the simulations consisted of 3D anatomical models of the LA, extracted from dynamic CT images acquired in five AF patients. All acquisitions consisting in ten volumes throughout the cardiac cycle were performed with the patients in sinus rhythm condition, triggered on the end of ventricular diastole.

Models with LAA were directly obtained from CT images as detailed in (29). In this study only the first volume (LA at end systole) was segmented to derive the patient-specific anatomical model. To create the 3D models with LAAO, we removed the LAA from the complete anatomical models previously obtained. To this purpose, the shape diameter function (SDF) was applied (30). Once the 3D SDF map was computed, the iso-contours on the LA meshes allowed a threshold-based segmentation. This segmentation resulted in anatomical regions with similar SDF values, identifying the pulmonary veins (PVs), the main body of the left atrium and the LAA. The anatomical position of the LAA (left side of the LA and below the PVs) was exploited to detect the LAA and to remove it (31). Manual correction of the LAA cut was applied in case the cut was too far from the pulmonary ridge.

Once the LAA was removed, we simulated the occlusion by generating a surface at the orifice. The closing surfaces were created by using MeshLab (32). Tetrahedral meshes had a size between 15 and 17⋅10^5^ finite elements (29, 31). The main geometric features of the devices as well as their fitting in the shape of the patient specific LAA ostium were taken into account. The simulated LAAO intervention was assumed to be correctly performed, thus emulating a complete occlusion without peri-device blood leakages and including the pulmonary ridge. The occluders based on the pacifier principle (28), characterized by a lobe and an additional disc to seal the ostium of the LAA from the left atrial side, were reproduced with a slightly concave closure. The screened Poisson surface reconstruction algorithm was instead used to reproduce the occluders based on the plug principle (28) to model also the convex structure of this particular LAA occlusion device.

To sum up, for each patient, we obtained a final set of three LA anatomical models: (1) the original model including the LAA; (2) the LAAO model obtained applying the pacifier principle; (3) the LAAO model obtained applying the plug principle.

**Figure 1:**
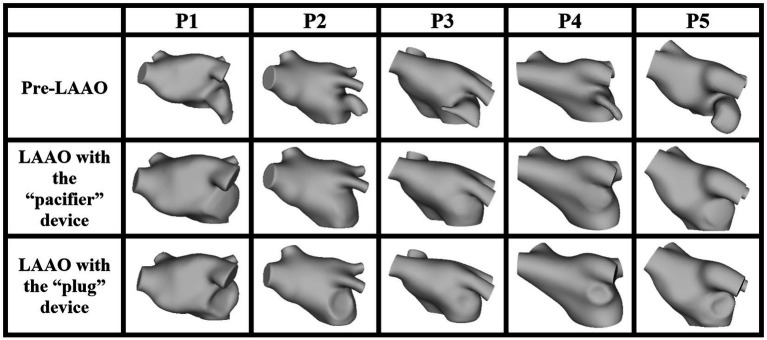
LA anatomical model, pre- (first row) and post-occlusion applying the devices based on the pacifier principle (second row) and on the plug principle (third row).

In Figure 1 we show the 15 anatomical models obtained in the five AF patients which represent the computation domain for the CFD simulations.

### 2.2. The computational fluid dynamics model

The CFD model used for the simulation of the hemodynamics of each anatomical model was the one detailed in (33) and based on the ℙ1–ℙ1 finite element method with SUPG-VMS stabilization of the Navier–Stokes equations described in (34). Details about blood flow modeling as a fluid governed by the incompressible Navier–Stokes equations written in the ALE frame of reference are reported in (35). The CFD model was previously modified for the specific application of LA blood flow simulation in AF patients in (29). To simulate contraction in AF conditions we employed a random displacement as described in (29) applied to the anatomical model previously obtained. As reported in (29), boundary conditions were set by adopting the mitral valve (MV) flowrate *F*^0^ described in (36). This flowrate was suitably modified for our application by removing the atrial contraction wave (A wave). Indeed, in AF condition, LA contraction is strongly reduced leading to a missing A-wave. At pulmonary veins (PVs), the flowrate was computed by enforcing mass balance conservation for all t ϵ (0,T]:


[1] $$F_{1}^{\mathrm{PV}}+F_{2}^{\mathrm{PV}}+F_{3}^{\mathrm{PV}}+F_{4}^{\mathrm{PV}}+F^{0}+\frac{\mathrm{dV}}{\mathrm{dt}}=0$$

where $F_{i}^{\mathrm{PV}}$ (*i* = 1,2,3,4) are the flowrates of each pulmonary veins, $F^{0}\;$is the flowrate at the MV section and $\frac{\mathrm{dV}}{\mathrm{dt}}\;$ is the flowrate associated to LA volume variation.

From Eq. 1, we defined $F_{\mathrm{tot}}^{\mathrm{PV}}$, the total flowrate at the PVs:


[2] $$F_{\mathrm{tot}}^{\mathrm{PV}}=F_{1}^{\mathrm{PV}}+F_{2}^{\mathrm{PV}}+F_{3}^{\mathrm{PV}}+F_{4}^{\mathrm{PV}}$$

The total flowrate $F_{\mathrm{tot}}^{\mathrm{PV}}$ through the four pulmonary veins was split with a criterion based on proportionality with their sectional area (29, 31):


[3] $$F_{i}^{\mathrm{PV}}=\frac{A_{l}}{A_{t}}F_{\mathrm{tot}}^{\mathrm{PV}}-F_{i}^{w}$$

where $A_{l}$ is the sectional area of each PV and $A_{t}$ is the sum of PVs sectional areas; $F_{i}^{w}$ is the flowrate due to changes in cross-sectional area of each PV orifice throughout the cardiac cycle. Indeed, in our specific application, $F_{i}^{w}$ is not null since we applied a random displacement function of small amplitude to our computational domain throughout the cardiac cycle (29). Therefore, PVs sections are allowed to move along the heartbeat. Consequently, for each time step, we were able to evaluate the varying flowrate at each PV to be applied into the computational model.

To avoid the presence of unphysical backflows which may give rise to numerical instabilities at the outflow boundary (i.e., MV), we considered the natural-type boundary condition reported in (37) with backflow penalization.

### 2.3. Numerical simulation and fluid dynamics parameter computation

For each patient, we performed a simulation in AF condition. AF was simulated by applying independently to each mesh vertex a random displacement of small amplitude. The aim of this random displacement function was to simulate atrial fibrillation during the cardiac cycle: in fact, contraction of the left atrium in AF is not synchronized and is strongly reduced in amplitude.

To avoid the influence of the unphysiological initial condition on fluid velocity, simulations were run for five cardiac cycles on the Galileo100 system [GALILEO100|SCAI (cineca.it)] using 1 node and 48 tasks per node. We reported the results of the fifth simulated cardiac cycle. Regarding the parameters of the fluid dynamics model, the time step was set to 0.005 s, dynamic viscosity was 0.035 poise, and the density was set to 1.06 g/cm^3^ (29).

Computed parameters able to describe LA fluid dynamics included LA velocity, particle washout and endothelial damage.

Blood velocity was the direct output of the CFD simulations.

Particle washout was assessed by populating the central part of the LA chamber with 20,000 massless particles at the beginning of the fifth cardiac cycle of the simulation and counting the residual number of particles within the LA at the end of the cycle [29].

**Figure 2:**
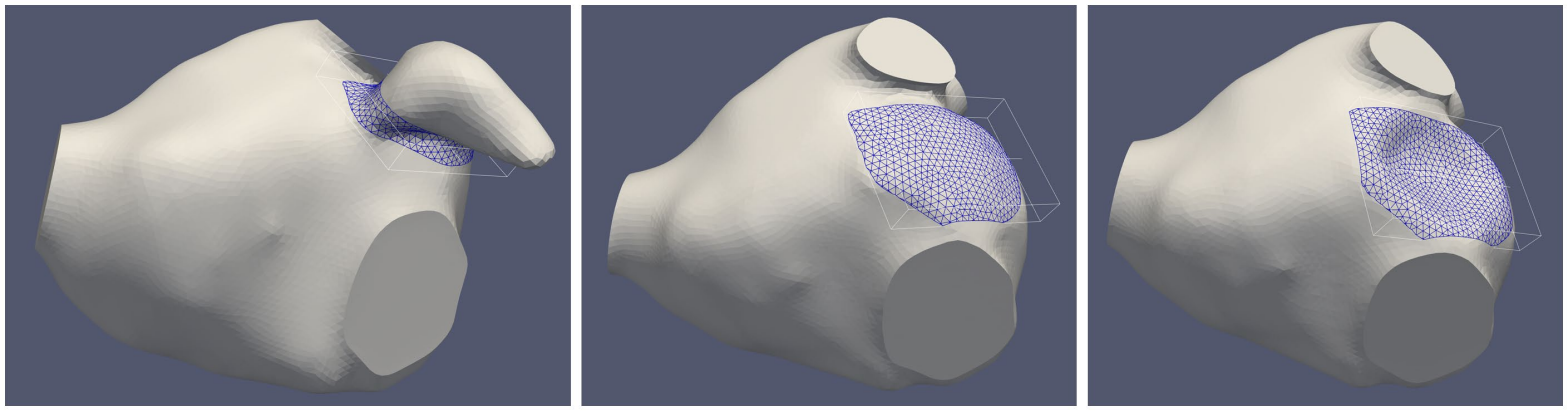
Selected volume near the surface of the implanted device in a representative patient before the occlusion (left panel) and after left atrial appendage occlusion (LAAO) with the “pacifier” device (middle panel) and with the “plug” device (right panel). The size of the volume in this patient was 1.54 cm^3^ (average volume in the five patients: 1.50 ± 0.29 cm^3^).

The endothelial cell activation potential (ECAP) was computed by the ratio of the oscillatory shear index to the time averaged wall shear stress (TAWSS) (24–27). ECAP values were normalized by the average TAWSS value computed within a volume surrounding the LAA ostium calculated from the pre-occlusion simulation (Figure 2). Regions prone to thrombogenesis are characterized by high ECAP values indicating large oscillatory shear flows and low wall shear stresses.

These parameters were evaluated within the entire LA model.

In addition, a local analysis was performed considering the same volume used for ECAP normalization populated with 2000 massless particles: the combined evaluation of blood velocity, particle washout and ECAP may support the quantification of thrombogenic risk in AF patients (38) and the assessment of DRT in the selected volume near the occlusion (25).

For a very preliminary assessment of peri-device leaks, we simulated a partial dislocation of the device after the closure intervention. The dislocation resulted in a leak of size 14.2 mm (long axis) and 5.05 mm (short axis). Results were reported in terms of blood velocity, particle washout and ECAP.

## 3. Results and discussion

The average time required for one simulation in each patient was 5 h and a half with a time-step of 0.001 s.

The trends and the changes due to the occlusion of the LAA in terms of blood flow velocity, particle washout and ECAP were similar among all the patients.

### 3.1. LA velocity analysis

**Figure 3:**
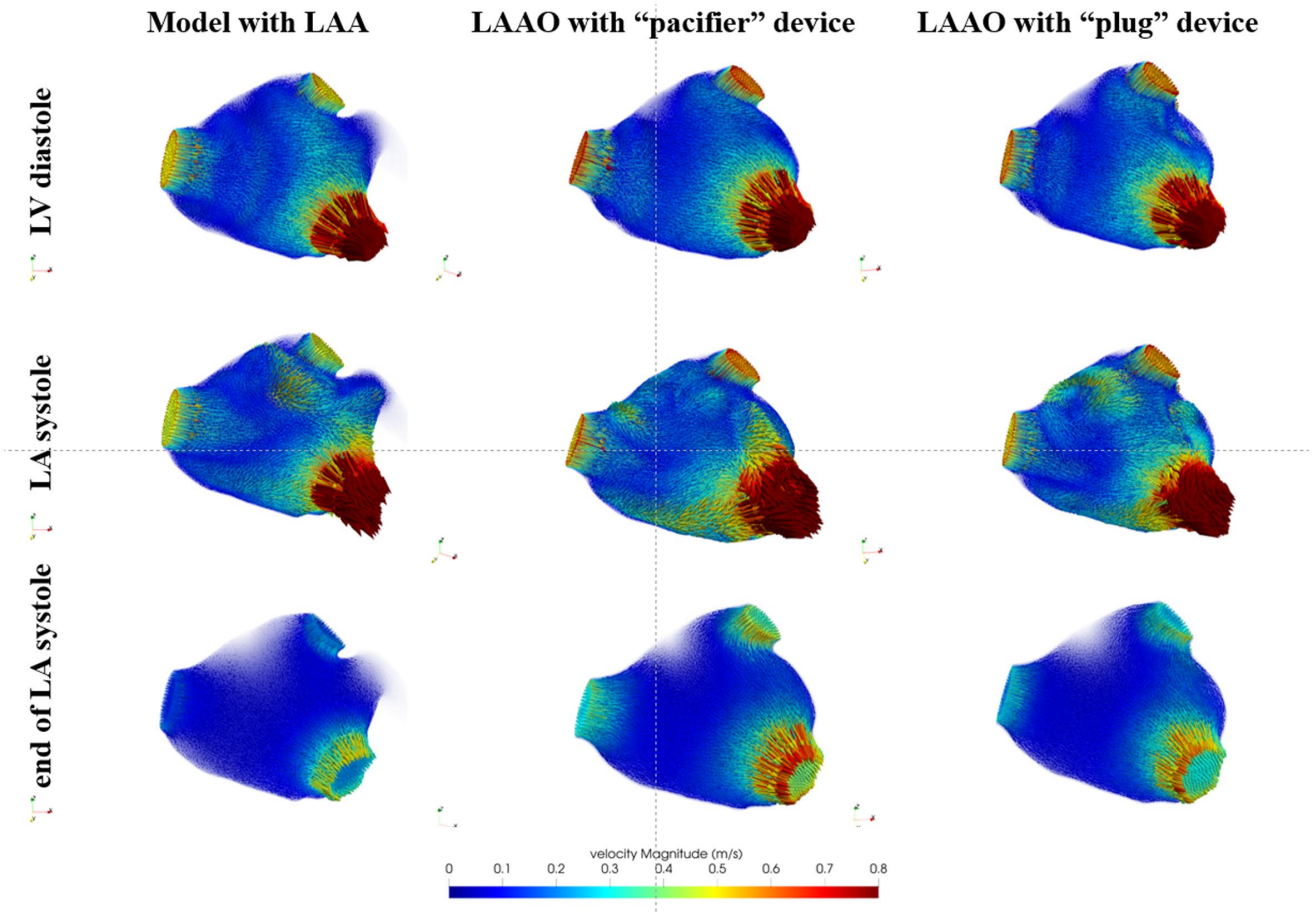
Blood flow velocity in the LA models acquired in a representative patient: in the first column we show the model with the left atrial appendage (LAA), in the second column the model after the occlusion with the device based on the pacifier principle and, in the third column, the model after the occlusion with the device based on the plug principle. First row refers to the beginning of ventricular diastole, second row to the atrial systole and the third row to the end of atrial systole.

In Figure 3 we show the computed velocity field for the LA model in one representative patient. The results of the CFD simulation in the LA with the LAA showed that, at the *beginning of ventricular diastole* (first row), the velocity at the MV reached the value of 0.8 m/s. Also, at the PVs velocities reached values between 0.4 and 0.6 m/s. Regarding the LAA, velocity vectors showed low amplitude and direction toward the LAA tip. For the LAAO simulations, we noticed that velocities at the PVs were higher with respect to the model with the LAA, with higher velocities recorded with the “pacifier” device compared to the “plug.” At the MV we observed similar values of the velocities with respect to the simulation with the LAA, with higher velocities within the LA chamber. Also, in these simulations, the direction of all the vectors seemed to converge toward the MV, with higher velocities with the “pacifier” device (second column) compared to the “plug” device (third column). Yet, it seemed that in the LAAO simulation with the “plug” device, the blood flow was less organized with respect to the LAAO with the “pacifier,” probably due to its convex shape that causes variations in the fluid streamlines direction. During *atrial systole* (second row), in the simulation with the LAA, velocity at the PVs decreased, and a velocity peak was observed on the anterior wall between the left superior PV and the LAA. For the LAAO simulations, higher velocities peaks were on the roof and in the distal parts of the LA chamber, close to the MV and the LAA ostium. In this phase the blood flow that was coming from the PVs moved toward the LAA ostium and collided with the occlusion plane. Therefore, the blood flow was constrained to go to the opposite direction with high velocities. Such situation was more pronounced in the simulation with the “pacifier” device that showed a more organized flow compared to the “plug” device in which higher velocities on the roof were found. At the *end of atrial systole* (third row), which was strongly reduced by the presence of AF, we observed lower velocities in correspondence of the MV in the simulation of the pre-occlusion LA. Inside the LAA, velocities remained very low (< 0.1 m/s), and we did not observe velocity vectors pointing toward the atrial chamber probably due to the AF condition and the strongly reduced LAA motion. Therefore, the risk of blood stagnation in the LAA could be considered high. As for the LAAO simulations, we observed higher velocity at the PVs and at the MV with higher values with the “pacifier” device.

In Table 1 the peak and average velocities at the MV and at the PVs are reported for all patients.

**Table 1:** Peak and average blood velocities at the mitral valve (MV) and pulmonary veins (PVs) in pre- and post-left atrial appendage occlusion (LAAO) simulations.

		P1	P2	P3	P4	P5	All patients (*m* ± SD)
Model with the LAA	MV peak velocity (m/s)	0.80	0.83	0.78	0.83	0.72	0.79 **±** 0.05
	MV average velocity (m/s)	0.56	0.44	0.42	0.45	0.41	0.46 **±** 0.06
	PVs peak velocity (m/s)	0.70	0.80	0.80	0.80	0.80	0.78 **±** 0.04
	PVs average velocity (m/s)	0.35	0.50	0.50	0.50	0.50	0.47 **±** 0.07
LAAO with “pacifier” device	MV peak velocity (m/s)	0.90	0.95	0.92	1.00	0.85	0.92 **±** 0.06
	MV average velocity (m/s)	0.47	0.47	0.46	0.48	0.44	0.46 **±** 0.02
	PVs peak velocity (m/s)	0.75	0.80	0.80	0.80	0.80	0.79 **±** 0.02
	PVs average velocity (m/s)	0.35	0.40	0.40	0.40	0.40	0.39 **±** 0.02
LAAO with “plug” device	MV peak velocity (m/s)	1.00	1.20	1.10	1.05	0.94	1.06 **±** 0.10
	MV average velocity (m/s)	0.50	0.52	0.50	0.49	0.48	0.50 **±** 0.01
	PVs peak velocity (m/s)	0.70	0.85	0.85	0.85	0.85	0.82 **±** 0.07
	PVs average velocity (m/s)	0.30	0.50	0.50	0.50	0.50	0.46 **±** 0.09

P: patient, m: mean value, SD: standard deviation.

Based on clinical literature (39) the mitral inflow velocity measured by echo Doppler in AF patients resulted in mean peak velocities between 0.8 and 1.3 m/s. These values are comparable with the maximum velocity values at MV in our simulations which range between 0.72 and 1.2 m/s (Table 1).

**Figure 4:**
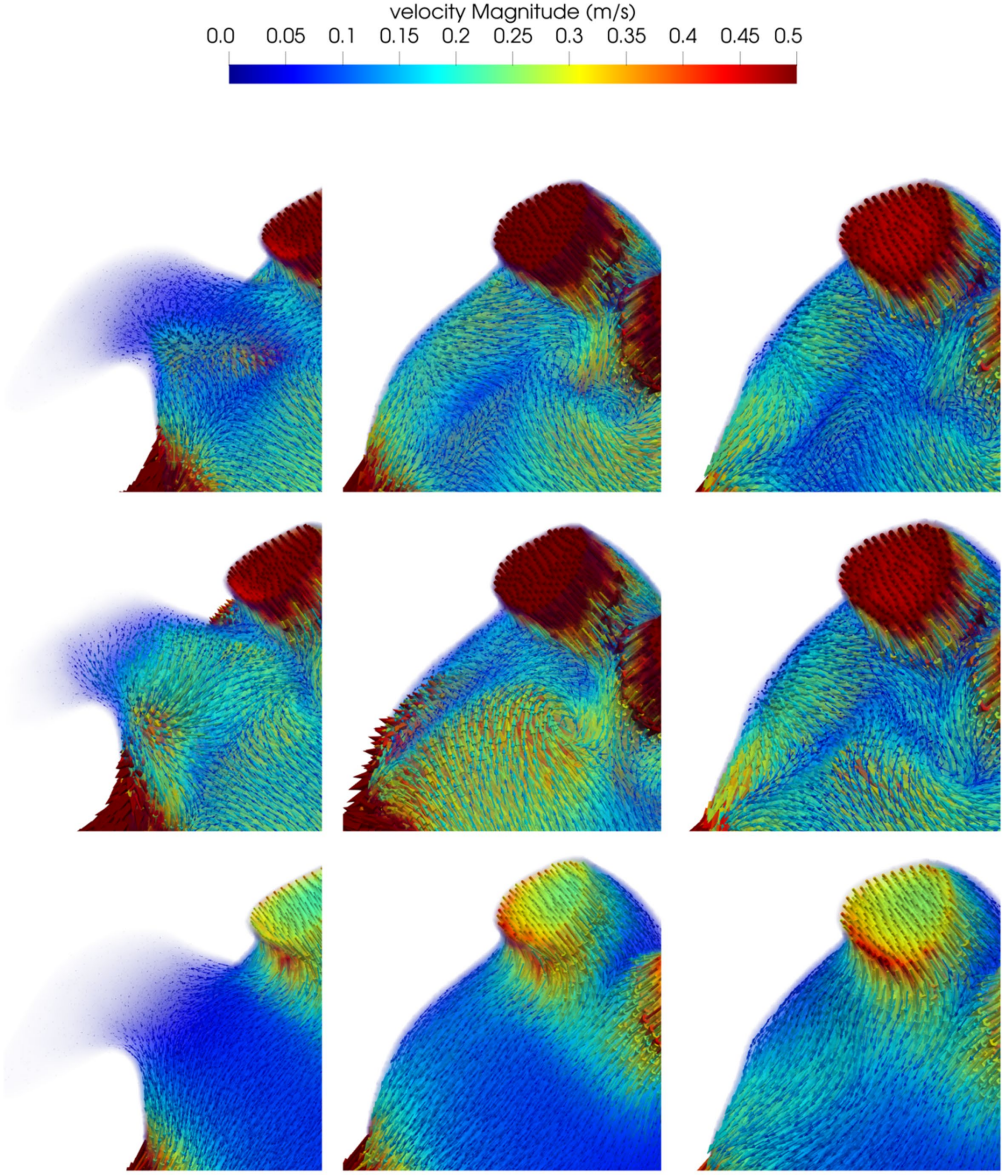
Blood flow velocity in the volume of interest close to the closure in a representative patient: in the first column we show the model before the occlusion; the model after the occlusion with the device based on the pacifier principle and the model after the occlusion with the device based on the plug principle are reported in the second and third column respectively. First row refers to the beginning of ventricular diastole, second row to the atrial systole and the third row to the end of atrial systole.

Blood flow velocities in the volume near the surface of the implanted device at the beginning of ventricular diastole (first row), during atrial systole (second row) and at the end of atrial systole (third row) are shown in Figure 4, post-occlusion with the “pacifier” device (first column) and with the “plug” device (second column). At the beginning of ventricular diastole with both devices, velocities pointed toward the MV; higher velocities and a more homogeneous flow was noticed with the “plug” device. During atrial systole velocities distribution caused by the contraction was more organized pointing toward the MV but with lower velocities; with the “pacifier” device the flow pointed toward the closure, making the outflow flow at the MV less effective. At the end of atrial systole, the flow at the closure was very organized with both devices but with higher velocities with the “plug” device.

The assessment of the mean blood velocities in the volume near the surface of the implanted LAAO device is reported in Table 2 for all patients. Velocities increased after the occlusion of the LAA, particularly during diastole; overall the simulations of the LAAO with the “pacifier” device showed higher velocities.

**Table 2:** Average velocities in a volume near the implanted device in the simulations performed in the five patients enrolled in the study.

Average velocities in a volume near the implanted device (m/s)									
	Model with the LAA			LAAO with the “pacifier” device			LAAO with the “plug” device		
	Systole	Diastole	Whole	Systole	Diastole	Whole	Systole	Diastole	Whole
P1	0.12	0.23	0.17	0.16	0.28	0.22	0.13	0.25	0.19
P2	0.18	0.26	0.22	0.17	0.29	0.23	0.18	0.33	0.25
P3	0.13	0.23	0.18	0.34	0.51	0.42	0.26	0.42	0.34
P4	0.15	0.23	0.19	0.15	0.30	0.23	0.14	0.25	0.20
P5	0.11	0.18	0.15	0.12	0.24	0.17	0.09	0.19	0.14

In the table we report the velocities during systole, diastole and throughout the cardiac cycle.

It is known from previous studies in literature (40) that LAA ostium velocities below 0.4 m/s in LA are associated with a higher risk of stroke, while velocities below 0.2 m/s are associated with the presence of thrombi within the LAA. In the pre-occlusion simulations, all the patients have velocities close to the threshold of 0.2 m/s, thus highlighting a potential thrombogenic spot. A possible reason for this behavior is the lack of LAA contraction due to the AF. Overall, in both LAAO models we recorded an increased average velocity; whilst in some patients the velocity was slightly higher, in other patients it almost doubled (Table 2). The effect of the occlusions affects more the diastolic phase.

### 3.2. Particle washout analysis

The analysis of the residual number of particles was performed for all patients enrolled in the study and observed that the simulations with the LAAO devices showed an improved washout of the atrial chamber as quantified by the residual number of particles: 392 ± 79 (LAA) vs. 254 ± 102 (“pacifier” device) and 294 ± 95 (“plug” device). These findings are also in agreement with studies in literature (21, 23) where the LA fluid-dynamics pre-and post-LAAO was investigated and authors found higher velocities close to the device and near the MV, in accordance with our results. These results were confirmed for the local analysis in which 20 ± 5 particles remained in the selected volume when the occlusion was performed with the “pacifier” device compared to the 26 ± 5 particles counted when the occlusion was performed with the “plug” device.

In all patients, a slightly more effective atrial washout in the “pacifier” configuration with respect to the “plug” configuration was confirmed because in this case the number of the residual fluid particles was consistently the lowest (see Table 3).

**Table 3:** Number (and percentage) of LA fluid particles remaining within the LA in the complete atrial chamber models (first row), for the LAAO simulation applying the “pacifier” device (second row) and for the LAAO simulation applying the “plug” device (third row); number (and percentage) of LA fluid particles remaining within the volume near the occlusion for the LAAO simulation applying the “pacifier” device (fourth row) and for the LAAO simulation applying the “plug” device (fifth row).

		P1	P2	P3	P4	P5
Entire LA chamber	Model with the LAA	510 (2.6%)	346 (1.7%)	310 (1.6%)	431 (2.2%)	365 (1.8%)
	LAAO with the” pacifier” device	372 (1.9%)	234 (1.2%)	124 (0.6%)	340 (1.7%)	198 (1.0%)
	LAAO with the “plug” device	414 (2.1%)	247 (1.2%)	197 (1.0%)	376 (1.9%)	236 (1.2%)
Volume near the occlusion	LAAO with the” pacifier” device	21 (1.1%)	17 (0.9%)	13 (0.7%)	24 (1.2%)	23 (1.2%)
	LAAO with the “plug” device	30 (1.5%)	24 (1.2%)	18 (0.9%)	29 (1.5%)	28 (1.4%)

P: patient.

### 3.3. Endothelial cell activation potential (ECAP) analysis

**Figure 5:**
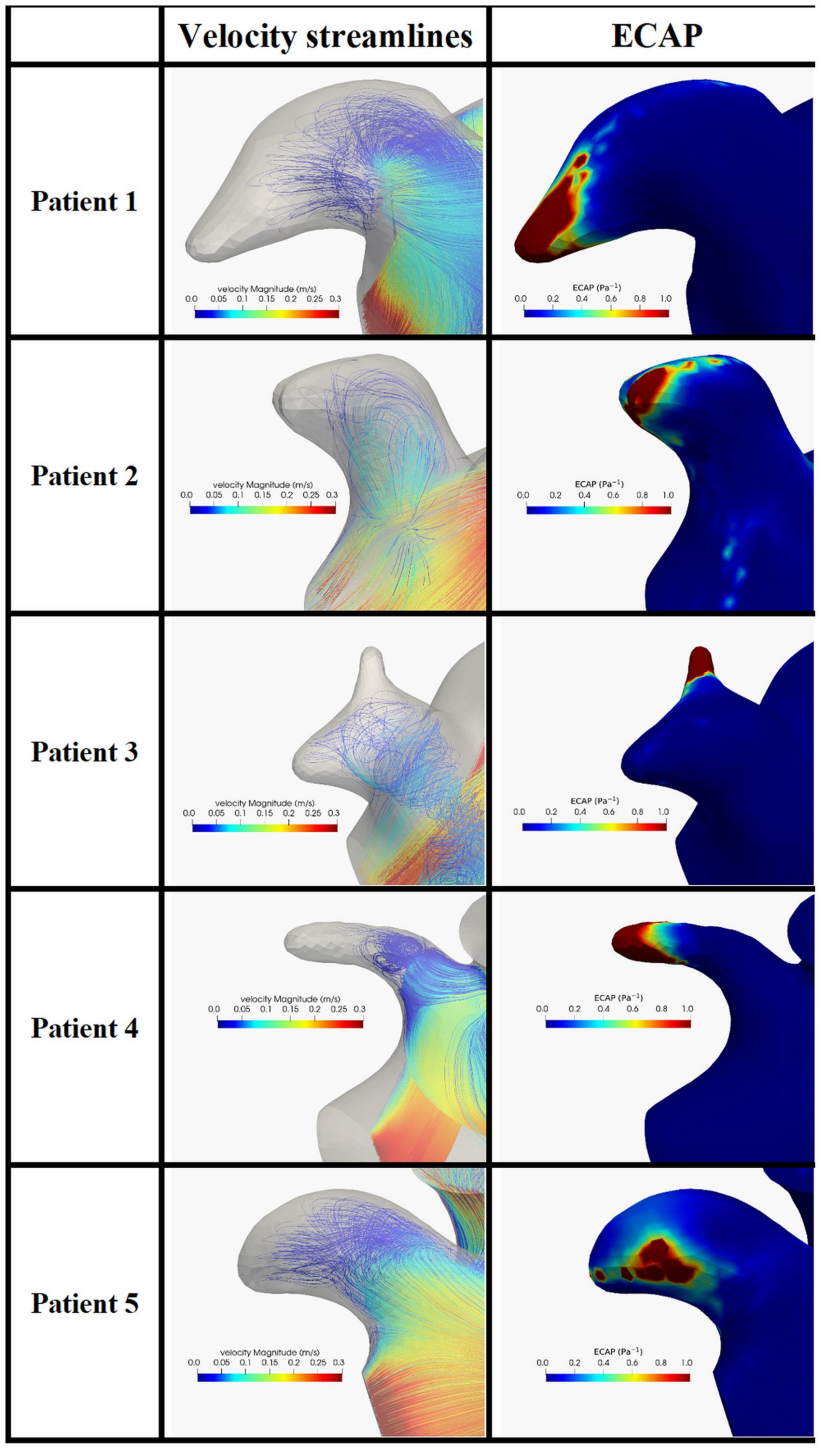
Blood velocity streamlines (first column) and ECAP map (second column) in the LAA and in the five patients enrolled in the study. ECAP maps were normalized considering the maximum ECAP value.

In Figure 5 we show the velocity streamlines and the ECAP values in correspondence of the LAAs of the five enrolled patients before the occlusion. In all the patients the distal part of the LAA showed very limited or absent blood velocity. The map of the endothelial cell activation potential near the device highlights regions with ECAP higher than 0.5 Pa^−1^ suggesting the presence of thrombogenic areas.

**Figure 6:**
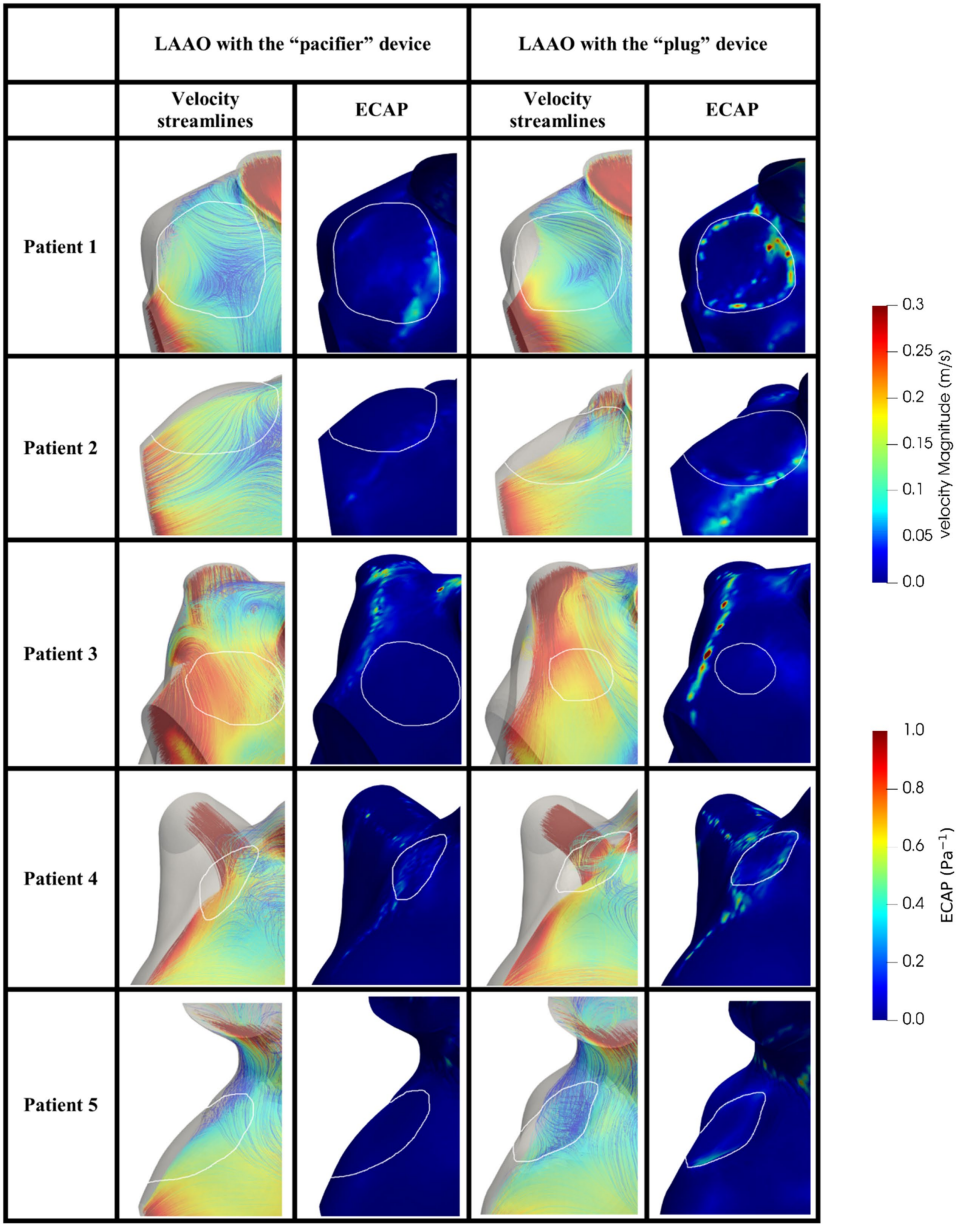
Blood velocity streamlines (first/third column) and ECAP map (second/fourth column) in the volume close to the occluder based on the pacifier/plug principle in the five patients enrolled in the study. White contours define the device location. ECAP maps were normalized considering the maximum ECAP value.

The same analysis was performed in the models after the occlusion applying the two devices. In Figure 6 we can appreciate higher velocities close to the occlusion with different distribution velocities among the two devices. In all patients, the ECAP spatial distribution shows that high-valued regions are smaller compared to the pre-occlusion simulations.

In agreement with other studies (25, 26, 38) we found velocities lower than 0.2 m/s and ECAP values higher than 0.5 Pa^−1^, which are both risk factors to predict DRT (Table 4); in our simulation the occlusion seems to reduce such risk by increasing blood velocity, reducing the ECAP maximum value; on note the size of the regions with high ECAP was also reduced (Figure 6).

**Table 4:** Max blood velocity and max ECAP value in the volume near the implanted device in the simulations performed in the five patients enrolled in the study.

	Model with the LAA		LAAO with the “pacifier” device		LAAO with the “plug” device	
	Max blood velocity (m/s)	Max ECAP (Pa^−1^)	Max blood velocity (m/s)	Max ECAP (Pa^−1^)	Max blood velocity (m/s)	Max ECAP (Pa^−1^)
P1	0.26	2.23	0.33	0.46	0.28	1.42
P2	0.31	1.85	0.35	0.28	0.39	0.61
P3	0.25	2.05	0.58	0.06	0.48	0.35
P4	0.25	2.31	0.32	0.13	0.29	0.58
P5	0.20	1.58	0.26	0.05	0.22	0.54

Comparing the “pacifier” and “plug” devices, the former ones seem to decrease the risk of DRT more efficiently than the latter ones (Table 4). Indeed, higher ECAP values are recorded along the border of the plug device, suggesting that blood clots may be found in between the LA wall and the curvature of the plug. These results are in agreement with the different device-related incidence of DRT and strokes reported in (28).

### 3.4. Peri-device leak simulation

**Figure 7:**
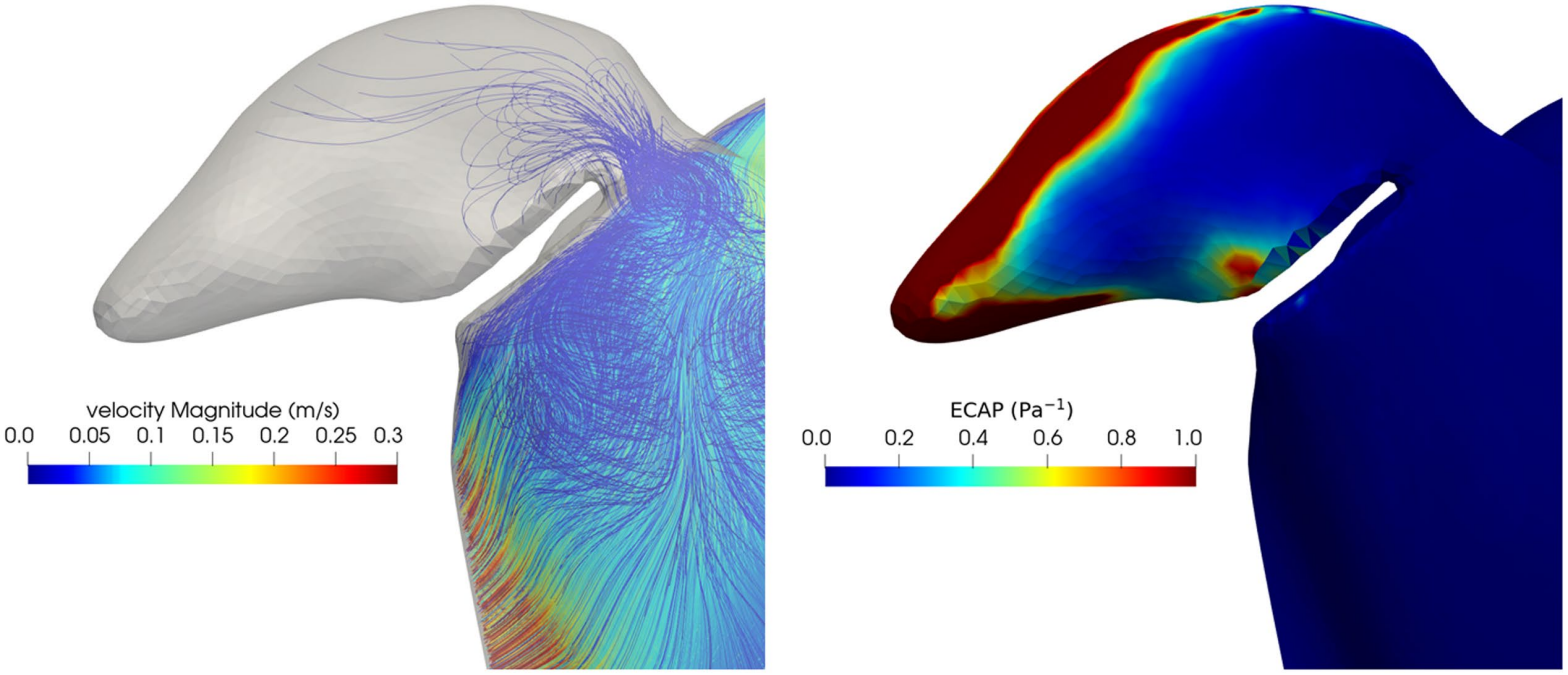
Blood velocity streamlines (left) and ECAP map (right) in the LAA and in the volume close to the occluders in the peri-device leak simulation.

The results of the peri-device leak simulation in one patient are shown in Figure 7. The presence of the leak allows the blood to enter and stagnate in the LAA. The ECAP map highlights a very broad high-valued regions of damage in the distal part of the LAA. Comparing the results of the peri-device leak simulation and the pre-occlusion one (Figure 5, first row) the DRT risk seems to be worsening due to a decreased maximum velocity (0.26 m/s pre-occlusion vs. 0.15 m/s leak simulation) and increased ECAP value (2.23 Pa^−1^ pre-occlusion vs. 3.84 Pa^−1^ leak simulation). The particle washout analysis with the dislocation resulted in 540 residual particles corresponding to 2.7%. Importantly, compared to the pre-occlusion simulation [35 (0.18%) particles inside the LAA], we found 84 (0.42%) particles in the LAA when the peri-device leak was simulated.

## 4. Conclusion

In this study a workflow for simulating the fluid dynamics effects of LAAO in AF was tested. Our simulations were feasible in all models derived from our small population. Preliminary results show the capability of this workflow to foresee consequences of LAAO in different scenarios (different type of occluders, different device locations, device related thrombosis and endothelial damage).

Our approach has several limitations. The simulation of the LAA occlusion without having the specific 3D model of the devices represent one of the main limitations of our study. The use of CAD models could better clarify the hemodynamic impact of the LAA occlusion in the left atrial chamber. We are also aware our results should be confirmed by running simulations including a higher number of beats to avoid the potential influence of the initial condition on fluid velocity, especially if the displacement field is a random field. On note, a preliminary test for one simulation run for ten cardiac cycles showed stable mean velocity profile inside the LA after the fifth cardiac cycle but further testing is required. In addition, it was shown (41) local wall stress assessment could be improved by including boundary layers; up to now we did not consider including boundary layers in our meshes. Moreover, the use of particles that do not interact with the wall nor between them to simulate blood stasis also represents a limitation.

In addition, our results could benefit from the application of a patient-specific LA motion field in AF. Unfortunately, due to ECG triggering requirements, up to date, quantification of such a motion field is not possible using MRI or CT imaging. Therefore, since AF contraction is qualitatively described as a disorganized and reduced motion of the LA, in our simulations we applied a random displacement field with small amplitudes in order to avoid also mesh degeneration. Once the patient-specific motion in AF is available, our pipeline would strongly benefit from such information.

An exhaustive validation of our approach would require 4D flow magnetic resonance imaging acquisition (42) which is rarely used in clinical practice due to the required expertise for a correct acquisition.

Despite such limitations, all our results consistently confirmed previous literature suggesting a more effective blood washout after LAAO and, consequently, a lower risk of blood clot formation. In addition, when comparing the two different types of devices a slightly improved thrombogenic risk is revealed when the pacifier-based occlusion is simulated. Our study could contribute to understand the fluid dynamics conditions leading to thrombogenesis and to identify the most effective devices in reducing the stroke risk for patient-specific morphologies of the LA. The presented framework might represent a step ahead toward the development of a better tool for the patient-specific thromboembolic risk assessment and preventive treatment in AF patients.

## Data availability statement

The raw data supporting the conclusions of this article will be made available by the authors, without undue reservation.

## Ethics statement

The studies involving human participants were reviewed and approved by Ethical Committee of the Romagna region (CEROM) n. 2323 Prot. 1276/2019. The patients/participants provided their written informed consent to participate in this study.

## Author contributions

ND derived the patient-specific model, contributed to data analysis and interpretation and drafted the manuscript. MF performed the simulations and computed the parameters, contributed to data analysis and drafted the manuscript. AM designed the study and performed the simulations. SS supported data analysis and interpretation. CC conceived and contributed to the design of the study, supported data analysis and interpretation, drafted the manuscript. All authors contributed to the article and approved the submitted version.

## Funding

This work was supported by the Italian Ministry of University and Research (Italian National Project, PRIN2017; ‘Modeling the heart across the scales’).

## Conflict of interest

The authors declare that the research was conducted in the absence of any commercial or financial relationships that could be construed as a potential conflict of interest.

## Publisher’s note

All claims expressed in this article are solely those of the authors and do not necessarily represent those of their affiliated organizations, or those of the publisher, the editors and the reviewers. Any product that may be evaluated in this article, or claim that may be made by its manufacturer, is not guaranteed or endorsed by the publisher.
